# How technical change has boosted fish aggregation device productivity in the Indian Ocean tuna fishery

**DOI:** 10.1038/s41598-023-45112-4

**Published:** 2023-10-19

**Authors:** Alex N. Tidd, Laurent Floc’h, Taha Imzilen, Mariana Tolotti, Laurent Dagorn, Manuela Capello, Patrice Guillotreau

**Affiliations:** MARBEC, Univ Montpellier, CNRS, Ifremer, IRD, Sète, France

**Keywords:** Ocean sciences, Conservation biology

## Abstract

Excess harvesting power can threaten the long-term sustainability of fisheries. Indicators of excess harvesting capacity must include input–output-based estimates of economic production efficiency. The increasing use of drifting Fish-Aggregating-Devices (DFADs) has boosted fishing productivity in high-seas tuna fisheries, perhaps beyond the biological capacity of the stocks, and is an object of global debate. We carried out a Data Envelopment Analysis (DEA) of relative changes in production efficiencies of the French purse-seine fleet targeting tropical tuna in the western Indian Ocean using two fishing strategies: (1) on floating objects (FOB) and (2) free swimming schools (FSC) using tuna catch and effort data spanning 1992–2019. We show that FOB fishing evolved dramatically through time with an estimated change of 3.6%yr^−1^ (8.0%yr^−1^ 2007–2019), in contrast to 2.1%yr^−1^ for FSC. While the efficiency level in combining and using inputs has barely changed for FOB fishing, it means that all the growth in productivity comes from technical change for this strategy. The dynamics is different for the FSC with a mixture of innovation and higher efficiency. Immediate plans to improve input-based management in this region are needed to prevent further risks of overfishing to yellowfin (*Thunnus albacares*) and skipjack (*Katsuwonus pelamis*) tunas.

## Introduction

World tuna fisheries and tuna-like species are an essential commodity, constituting on average ~ 8% (7.8 million tonnes) of globally traded seafood and ~ 20% of the total value of all marine capture fisheries^1^, with an estimated consumption rate of 0.45 kg per capita per year (canned tuna) (2.2% of the global fish consumption)^2^. The growing demand for tuna species and their high value make them subject to increasing fishing pressures^3^. With the human population growing at a rate of 1.6% per year^4^ and the demand for high-protein food continuously rising^5^, fishing fleets respond by increasing in size and efficiency^6^. The resulting overfishing problem^7,8^, combined with the adverse impacts of climate change^9^, poses severe risks to the social and economic well-being of many countries^10,11^.

### The increasing role of FADs in the expansion of IO tuna fisheries

**Figure 1:**
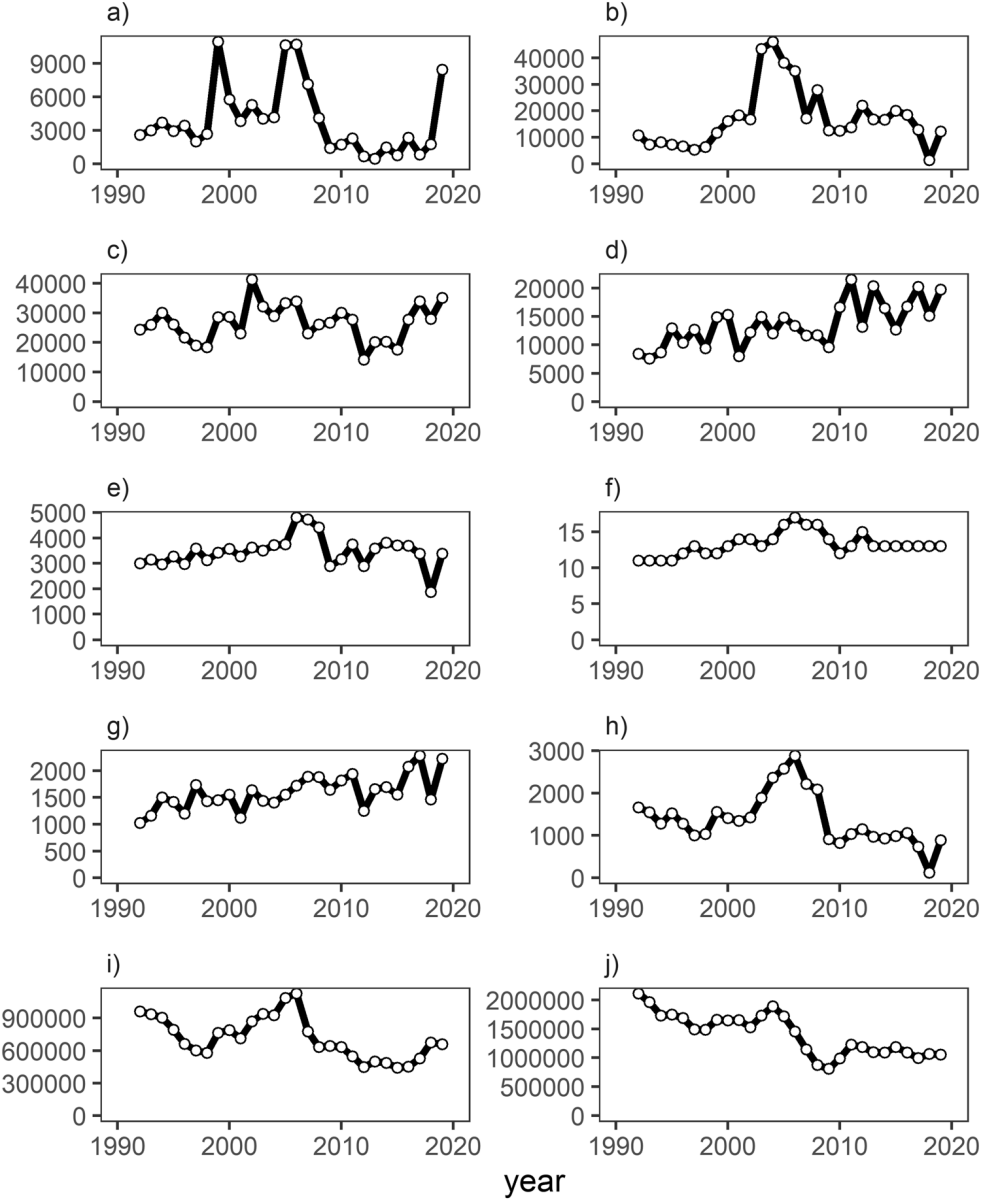
Summary plots of French PS fishing in the Indian Ocean 1992–2019 (**a**) free- school skipjack catch (tonnes), (**b**) free-school yellowfin catch (tonnes), (**c**) floating object skipjack catch (tonnes), (**d**) floating object yellowfin catch (tonnes), (**e**) number of days at sea, (**f**) number of PS vessels, (**g**) number of floating object sets, (**h**) number of free school sets, (**i**) skipjack biomass (tonnes) IOTC and (**j**) yellowfin biomass estimates (tonnes) IOTC.

The Indian Ocean (IO) hosts one of the most essential tuna fisheries in the world. It differs from other oceans worldwide by a large proportion (~ 50%—IOTC-2022-WPTT24-03a_Rev1) of tuna caught by small-scale fishers and the prevailing share of catches coming from areas beyond national jurisdiction. Tuna catches are split between non-industrial and industrial fleets, supporting many cultures and economies. Non-industrial fleets are characterised by coastal artisanal vessels operating in their Economic Exclusive Zones and using gillnets, pole and line, hand lines, etc. On the other hand, industrial fleets are usually represented by distant water fishing nations, consisting mainly of purse seine (PS) and longline vessels^12^. The most predominant catches in the IO are two tuna species, yellowfin (*Thunnus albacares*) and skipjack (*Katsuwonus pelamis*) tunas (see Fig. 1). Yellowfin and skipjack tunas are two of the most valuable fisheries in the world, with an end value worth $US15.8Bn and $US16.1Bn, respectively^3^. Both species are caught in significant quantities by purse-seiners utilising free school and associated fishing strategies. Free school fishing involves fishing on a free-swimming school of tuna, localised by fishers when the fish are feeding at the surface. At the same time, fishing on associated schools exploits floating objects (FOBs) to locate tuna aggregations beneath them. For both fishing modes, the tuna schools/aggregations are caught by encircling them using a PS net on the surface and subsurface of the ocean. Each of these operations is an un-associated or associated ‘set’. FOBs can be natural (e.g. logs) or artificial Fish Aggregating Devices (FADs). To track their drifting movements and check remotely the associated biomass underneath, purse seiners equip the FADs (and occasionally, the natural logs that they encounter) with instrumented buoys, which represents a significant innovation in high-seas fishing^13–15^.

### DFAD use and fishing efficiency

DFADs are essentially artificial floating structures consisting of metal frames or bamboo rafts deployed by fishers in the open ocean to target associated schools of tuna^16^. Depending on the DFAD design, they can present a vertical substructure stretching an average of 50 m below the surface or be fully submerged. Since their development in the 1990s, there has been a “massive” expansion in the usage of DFADs by the industrial PS fleet globally^14,17^. All types of FOBs are known to attract large quantities of tuna, and the reasons and mechanisms of this associative behaviour remain unknown to scientists^12,18^. Since the 2000s, the DFADs have evolved to be far more sophisticated due to the development and advancements of GPS buoys equipped with echo-sounders^13,19–21^. These new technologies substantially increase the fishing efficiency of PS vessels^22,23^. Simultaneously, the massive use of DFADs raised several concerns about their ecological impacts^24^. Compared to FSC, DFAD fishing implies higher catches of juvenile yellowfin and bigeye tunas^25^ and increased bycatch of vulnerable species such as oceanic sharks^26^.

Furthermore, DFADs contribute to marine debris and can threaten sensitive habitats through stranding^27,28^. Finally, depending on the ocean, specific resolutions imposed the use of non-entangling FADs since the use of underwater nets beneath DFADs has been shown to cause the ghost fishing of turtles and sharks (e.g., silky shark: *Carcharhinus falciformis*^29^). The combined effect of increased DFAD use can improve fishing efficiency, reduce fishing effort, and maintain or raise catch rates on the target tunas even when stocks decline^30^. However, a recent study has shown that the increase in DFAD deployments over time led to increased densities in specific areas that have resulted in a decrease in catch per unit effort (cpue) due to tuna's biomass being fragmented between DFADs^31^. In contrast, the success rate of FSC sets is lower even though this fishing activity has adopted modern equipment to detect tuna concentrations using high-powered binoculars and bird radars^13^. The FSC fishing operation requires a high degree of skill by the skipper and crew to catch the fast-swimming schools of tunas but tends to yield a higher valued yellowfin tuna due to the larger fish size compared to the smaller size of fish associated with DFADs^32^.

Within fisheries production, differences between catch yields for the same level of effort can be attributed either to technical innovations (e.g., technological advancements in DFADs, global positioning systems and sonar), species abundance or better use of available inputs (e.g. through higher skipper and crew skill). Fishers constantly strive to improve their productivity, resulting in distinct physical and economic efficiencies among fishers. Fishing inefficiency can contribute to an excess of fishing capacity (or underutilised fishing effort), which can jeopardise economic profitability and the sustainability of the fishery.

### Management measures to rebuild yellowfin stocks in the IO

Tuna stocks are monitored and managed via a set of conservation and management measures under the authority of the Indian Ocean Tuna Commission (IOTC). The IOTC fisheries governance is mainly based on single targeted species stock assessments and the resulting IOTC conservation and management measures (CMM) to reduce impacts on the stock of concern, and also the retention of bycatch, entangling FAD bans, and more general effects on the ecosystem (e.g., use of biodegradable FADs). For example, IOTC adopted the Resolution 16/01 which implemented a stock rebuilding plan for yellowfin via a Total Allowable Catch (TAC) level to cap the catch within biologically safe limits. This resolution was followed by other CMMs (Res 17/01, Res 18/01, Res 19/01, Res 21/01) that further redefined the levels of the TAC. Nevertheless, in 2021, the yellowfin tuna stock assessment estimated that the stock status of yellowfin tuna was still overfished and subject to overfishing (see IOTC–2021–SC24–R[E]_Rev1, 2021). Overfished means that the spawning stock biomass in the water is less than the biomass needed to produce at maximum sustainable yield (MSY), and the term ‘subject to overfishing' implies that the fishing mortality (i.e. the adequate level of fishing effort) is greater than that to produce MSY. A similar diagnosis was made for bigeye tuna (*Thunnus obesus*) at the 27^th^ IOTC annual meeting, resulting in the first TAC set for bigeye tuna in 2024 (https://iotc.org). In the same ocean, the most recent stock assessments conducted for skipjack tuna determined that its stock status is not overfished or subject to overfishing (IOTC–2021–SC24–R[E]_Rev1, 2021). With the yellowfin tuna stock still in a rebuilding phase, many environmental stakeholders argue that the TAC has primarily failed and needs to be significantly reduced along with improved management of DFADs (resolutions reducing the use of DFADs Res. 12/08, 15/08, 17/08, 19/02, and proposal IOTC-2021-SS4-PropD).

### When efficiency gains jeopardise the future of IO tuna fisheries

This paper aims to improve our understanding of the French PS fleet IO efficiency dynamics via a new set of indicators to provide helpful insight into fishery input and output relationships. Furthermore, this study targets a better quantification of the contribution of DFADs to fishing productivity by separating efficiency gains, i.e. a better use of available inputs or the need to reduce them for an identical output level, from technical change. We demonstrate that technological change has been the key driver of IO PS productivity to the extent of jeopardising the fishery's future. It enhanced the fishing capacity beyond the biological limits of some tuna stocks in the IO, particularly yellowfin and bigeye tunas.

Primarily, we apply a Data Envelopment Analysis (DEA—a non-parametric method) for a time series spanning 1992–2019 for the French PS fleet fishing both on FOBs and FSC to estimate technical efficiency (TE). TE is an economic term which means the observed level of what is caught (output) relative to what could be potentially caught from a given set of fixed (e.g. vessel engine power, gross tonnage, length) and variable inputs (days at sea, the number of FAD and FSC sets) that would be used at their maximum efficiency^6^. TE refers to the relative effectiveness of a fisher (vessel) to use and combine its inputs when producing an output, with regard to the production frontier corresponding to the most efficient vessels of the fleet. Using a DEA linear-programming methodology, TE scores between 0 and 1 are calculated for each vessel on the basis of the distance to the production frontier. A vessel on the frontier is thus said to be efficient (TE score = 1), and vessels lying below the frontier are less efficient (TE score < 1). In a separate model (described above), we also estimate a set of capacity utilisation scores (CU) using a similar method. CU is defined as the ratio of actual to capacity output (i.e. the production frontier) corresponding to the maximum catch level with the existing amount of fixed inputs. Vessels fishing with the same fixed inputs should have the same value of CU. A CU index smaller than 1, showing a capacity under-utilisation, often reveals an excess capacity in a fishery and indicates the magnitude of this excess. If the CU scores between two identical vessels (e.g., same length and power) are different, it could be due to exogenous (random) events affecting differently their catch, such as piracy events, changes in market and cost conditions^33,34^. However, other factors that may affect CU include stock abundance, density, and the TE, e.g., the skipper's and crew's skill^30^.

To obtain a better (unbiased) estimation of the excess capacity, we must therefore correct the CU by the TE score. The differences in CU (underutilisation) can be adjusted if the TE (inefficiency) is known. The ratio of the two scores (CU/TE) is derived as an unbiased capacity utilisation score (UCU) and represents underutilisation (or excess capacity) rather than random noise. These above scores cannot be measured with absolute certainty due to changes in technology and economic conditions^35^, and as such, have to be estimated as a comparative process of individual vessels within a season/year from a time series of fishing effort (input) and catches (output). This is why Malmquist indices are developed as a measure of total factor productivity change (PC) between two periods (e.g. 2018 and 2019) and throughout the whole sample period, disentangling the actual TE effect from the technical change effect^30^. The indices can be actually decomposed into efficiency change (EC), or the change in productivity due to the difference in the use of inputs (e.g. skipper skill, faster sets to avoid fish escapement, better use of existing capacity, learning effect), and technical change (TC), which is the change in productivity due to novel technology (i.e. FADs equipped with echo sounders, the assistance of supply vessels for example), and then to derive an estimate of ‘effort creep’ or percentage year on year change in TC. In other words, greater efficiency means that a vessel is getting closer to a fixed production frontier, while technical change corresponds to higher yields explained by the shift of the production frontier itself for all vessels.

Finally, to understand what factors affect UCU, a linear regression with covariates likely to affect the TE of fishing vessels is fitted. The choice of covariates is based on the DEA literature applied to fisheries and includes fuel prices^36^, vessel kW power, annual DFAD deployments^14,23^, threats of piracy events^37^, and the Indian Ocean Dipole index (DMI). The latter is considered because different positive/negative phases can significantly alter the abundance and availability of tropical tunas due to a regime shift of the thermocline, food availability, primary production and the sea surface temperature^38^. We then assess the specific impact on UCU for each covariate and evaluate the effect of a 1% increase in vessel kW power, for instance (and other covariates), on the subsequent % change in UCU.

## Results

### Estimation of UCU

**Figure 2:**
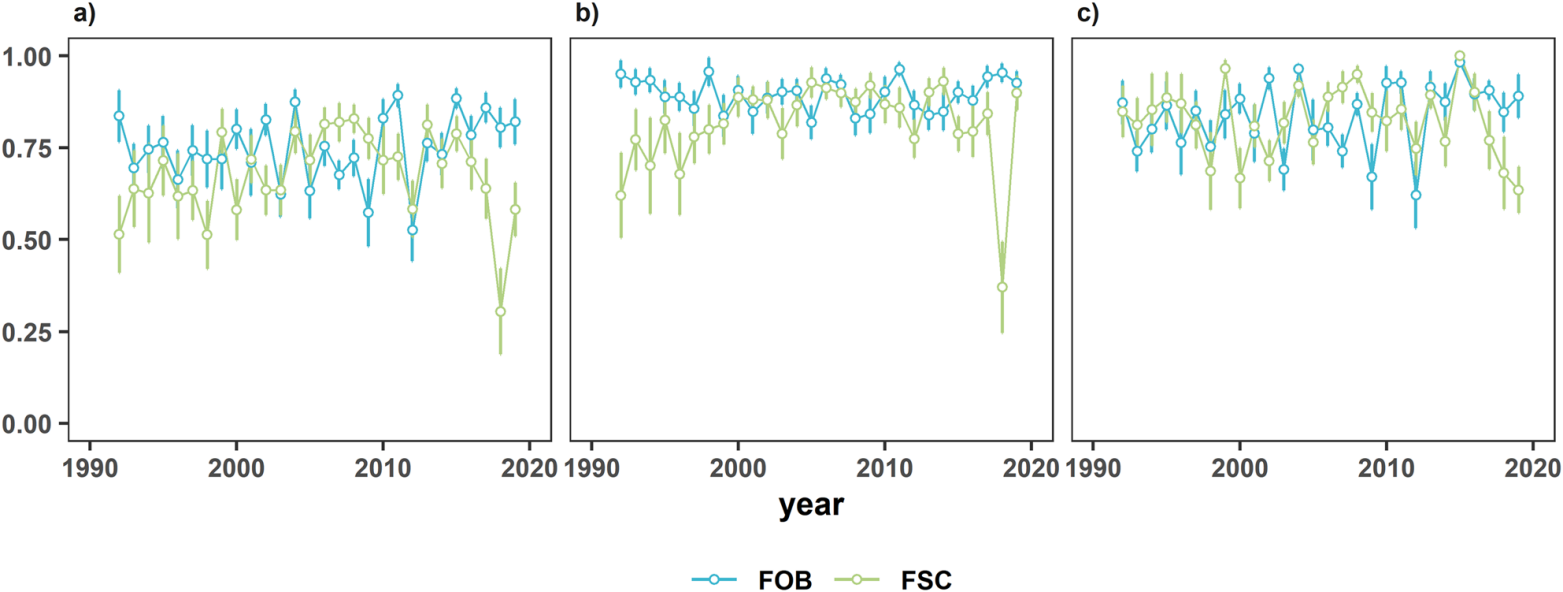
(**a**) Average capacity utilisation (CU), (**b**) average technical efficiency (TE), (**c**) average unbiased capacity utilisation (UCU), all plots with standard error bars.

The results from the DEA are presented in Fig. 2. The scores for (a) Capacity Utilisation (CU), (b) Technical Efficiency (TE), and (c) Unbiased Capacity Utilisation (UCU) averaged across vessels within a year by FSC and FOB show differing trends. For the FOB CU, the earlier years of the study period suggest a decline from ~ 0.8 in 1992 to 0.60 in 2009. However, an increasing trend is evident from 2009 to the end of the time series, other than a dramatic fall in 2012. For example, CU in 2012 was 0.50 from a time series high of 0.80 in 2011. Observations from the time series for FOB TE display a high and stable technical efficiency estimate of ~ 0.8 throughout the time series. The UCU results imply, for example, that this fleet could have caught ~ 25% (UCU = 1/0.80) more fish with such a fleet capacity, or caught the same amount of fish with ~ 20% fewer vessels (UCU = 1–0.80). For the free school sets, TE (average score of 0.6) and CU (average score of 0.5) initially display a degree of inefficiency in 1992 but gradually show an increasing trend up to 2008, where they plateau at ~ 0.8 for CU and TE at 0.8 in 2006. Apart from a high > 0.8 score in 2014, the fishing performance declined to a time-series average low score in 2018 of 0.4 (TE) and 0.3 (CU), respectively. The UCU trend also matches the CU, but UCU shows, on average, a higher score of ~ 0.75 throughout the study period compared to ~ 0.65 for CU.

### Examining changes in efficiency—Malmquist indices

**Figure 3:**
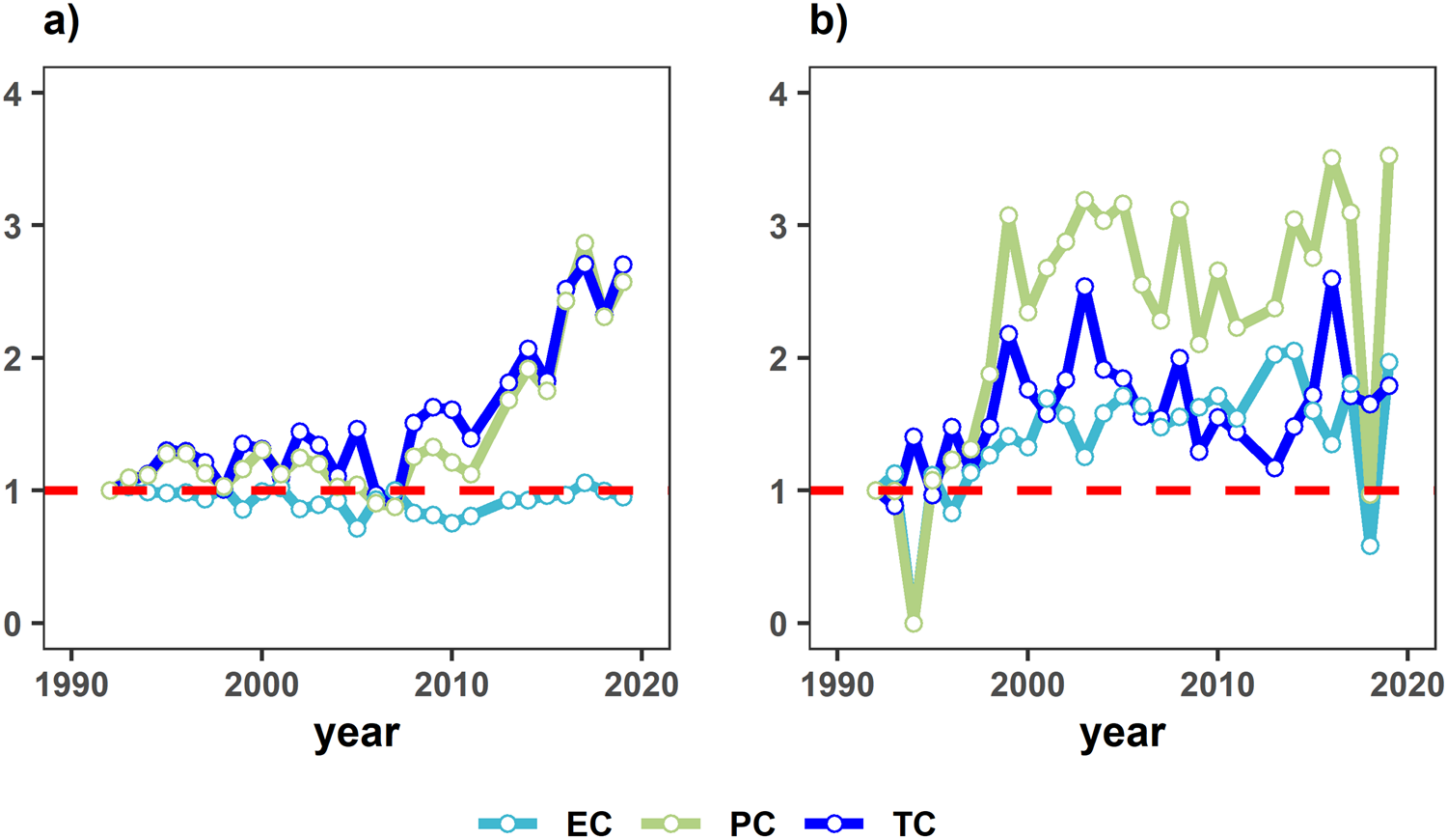
(**a**) Cumulative change (1992 = 1) floating object Malmquist indices (geometric means), (**b**) cumulative change (1992 = 1) free school Malmquist indices (geometric means). EC = Efficiency Change, TC = Technical Change, and PC = Productivity Change.

The individual vessel data of productivity change (PC) and corresponding decompositions of technical change TC and EC efficiency change as geometric means between 1992 and 2019 by the fishing strategy are presented in Fig. 3 as cumulative multiplicative inter-annual changes. For example, the PC value of ~ 3 for the FOB and FSC strategies indicates that the fleet is over three times more efficient in 2019 than in 1992. In terms of the EC and TC for both fishing strategies, these depict some exciting patterns. For example, PC for FOBs is entirely driven by TC. The TC is most pronounced between 2007 and 2019, with an overall change throughout 1992 and 2019 of + 3.6% annually.

In contrast to this upward frontier shift, EC has remained relatively stagnant, around the relative value of 1. This value for EC would reflect an inability to produce as much as possible given the fleet's current technology and possible homogeneous performance. In stark contrast, for the FSC PS, the EC showed gradual progression until 2013/2014, when a decline was observed. Furthermore, TC also showed continual improvements until ~ 2007/2008 and then a sudden decrease for seven years and picked up around 2015/2016 before falling again and stabilising for the last few periods with an estimated TC of 2.1% year on year. The sudden fall in FSC EC in 2018 is due to a fall in fishing effort (See Fig. 1). The PC, on the other hand, for FSC has been significantly high on average with a cumulative value of over two (+ 2% annually) for most of the time series, barring the initial period, 1992–1998, and 2018, where the value falls below the relative value of one (see number of sets/catch in Fig. 1).

### Factors affecting UCU

A ‘candidate’ model was chosen to examine factors that could explain vessel-specific differences in UCU among five of the 'best' models obtained by the selection algorithm (see R Package glmulti, Calcagno and Mazancourt 2010). The AICs were sorted from lowest to highest by fishing strategy, with the lowest AIC score selected as the candidate model (Table 1). Diagnostics from models were checked via graphs for distribution of the residuals and checked for autocorrelation and heteroskedasticity. Auto-correlation was not found to be present, but a degree of heteroskedasticity was observed in all candidate models. The candidate models were bootstrapped 1000 times to minimise the heteroskedasticity symptom, the parameter estimate was bias-corrected, and the significance was re-calculated.

**Table 1:** Candidate model diagnostics.

	df	AIC	Delta AIC
**UCU_FOB 1992–2019**			
UCU ~ 1 + TAC + fadsets + fuel + events + yftpr + kW	8	− 336.92	0.00
UCU ~ 1 + TAC + fadsets + fuel + events + yftpr + kW + dmi	9	− 335.98	0.94
UCU ~ 1 + fadsets + fuel + events + yftpr + kW + dmi	8	− 334.61	2.31
UCU ~ 1 + fadsets + fuel + events + yftpr + kW	7	− 334.03	2.89
UCU ~ 1 + TAC + fadsets + fuel + events + yftpr + dmi	8	− 331.73	5.19
Null	2	− 62.64	274.29
**UCU_FSC 1992–2019**			
UCU ~ 1 + fscsets + events + yftpr + kW + dmi	7	− 173.44	0.00
UCU ~ 1 + TAC + fscsets + events + yftpr + kW + dmi	8	− 172.17	1.27
UCU ~ 1 + fscsets + fuel + events + yftpr + kW + dmi	8	− 171.79	1.64
UCU ~ 1 + fscsets + events + yftpr + dmi	6	− 171.27	2.16
UCU ~ 1 + fscsets + yftpr + kW + dmi	6	− 170.61	2.83
UCU ~ 1 + TAC + fadsets + fuel + events + yftpr + kW + dmi	9	− 170.48	2.95
Null	2	− 20.73	152.71
**UCU_FOB 2013–2019**			
UCU ~ 1 + fadsets + kW + buoys	5	− 117.66	0.00
UCU ~ 1 + fadsets + yftpr + kW + buoys	6	− 116.30	1.35
UCU ~ 1 + fadsets + events + kW + buoys	6	− 115.84	1.82
UCU ~ 1 + fadsets + fuel + kW + buoys	6	− 115.78	1.88
UCU ~ 1 + fadsets + kW + dmi + buoys	6	− 115.77	1.89
UCU ~ 1 + fadsets + fuel + events + yftpr + kW + dmi + buoys	9	− 111.67	5.99
null	2	− 85.16	32.49

*df* degrees of freedom; *AIC* Akaike Criterion Score.

#### UCU_FOB model 1992–2019

For the UCU_FOB candidate model over the period 1992–2019 presented in Table 2, we found the number of FOB sets, piracy events, yellowfin tuna real price per tonne (constant USD of 2015), and fuel real price (constant USD of 2015) to be highly significant (*p* < 0.001). In contrast, the power of vessels in a particular year and the introduction of TAC was significant at the 5% level. In terms of interpretation of the coefficient signs of the variables, negative signs were observable for TAC, piracy events, yellowfin real price and vessel power, which implies a negative influence of these variables on UCU in contrast to the number of FOB sets and fuel prices, which depict a positive influence on UCU. The calculated elasticities in Table 2 (the expected change in UCU with a 1% change in the individual explanatory variable) indicated that for a 1% change in the number of FOB sets, the corresponding UCU would increase by 0.52%. For example, an increase of 1% in the yellowfin price would result in a 0.2% decrease in UCU.

**Table 2:** UCU by fishing strategy.

	Estimate	Bias	Std. error		Elasticity
**UCU_FOB 1992–2019**					
(Intercept)	0.625115	0.0030004	0.0616486	***	
TAC	− 0.061742	0.0013473	0.0291310	*	
fadsets	0.003603	− 0.0000015	0.0002477	***	0.52
fuel	0.000162	0.0000013	0.0000448	***	0.12
events	− 0.000347	− 0.0000013	0.0001008	***	− 0.04
yftpr	− 0.000094	0.0000007	0.0000240	***	− 0.2
kW	− 0.000026	− 0.0000010	0.0000106	*	− 0.14
*Model R*^*2*^	0.54	0.006	0.035		
**UCU_FSC 1992–2019**					
(Intercept)	0.463671	− 0.0034650	0.0849104	***	
fscsets	0.002668	0.0000120	0.0002234	***	0.34
events	0.000229	− 0.0000002	0.0001034	*	0.02
yftpr	0.000083	− 0.0000004	0.0000223	***	0.18
kW	− 0.000024	0.0000005	0.0000136		− 0.13
dmi	0.170094	0.0024508	0.0576198	**	0.02
*Model R*^*2*^	0.36	0.007	0.07		
**UCU_FOB 2013–2019**					
(Intercept)	1.166138	0.0024519	0.0878038	***	
fadsets	0.001892	− 0.0000235	0.0004470	***	0.29
kW	− 0.000099	0.0000004	0.0000247	***	− 0.53
buoys	− 0.000065	− 0.0000005	0.0000272	*	− 0.05
*Model R*^*2*^	0.32	0.01	0.07		

Coefficient estimates for (OLS) and bootstrapped OLS (bias). Statistical significance at ‘***’ 0.001 ‘**’ 0.01 ‘*’ 0.05 ‘.’ 0.1 and bias-corrected elasticities.

#### FSC model 1992–2019

Examining key variables resulting from the FSC model 1992–2019 (Table 2), significant positive coefficients included the number of free school sets, the number of piracy events, the positive dipole mode index and yellowfin tuna price contrasted with vessel power being the only slightly significant negative coefficient. The variable for the number of free school sets has the highest value in terms of elasticity resulting from a 1% change, resulting in a 0.34% change in UCU.

#### FOB model 2013–2019

The FOB model 2013–2019 omitted the TAC variable due to having a highly significant positive correlation with the number of DFADs deployed. This part of the study aimed to see the effect of the number of DFADs deployed. Here, we find that the coefficient for the number of buoys deployed negatively affected UCU and vessel power. In contrast, the number of FOB sets is the only positive factor influencing UCU (Table 2), i.e. with an elasticity of 0.29% increase (decrease) in UCU for every 1% increase (decrease) in the number of FOB sets.

## Discussion

The scarcity of fish resources resulting from climate change, over-harvesting and human population growth poses severe risks to economic and social well-being^5,39^. To achieve sustainable fisheries management strives to balance fishing opportunities, i.e. the set quantity of catches from each stock for all sectors of a wider fishing community (ensuring the social and economic viability of coastal communities, maintaining fisher knowledge and providing fish products for broader society) with the need to maintain fish stocks in a healthy state^40^;

Our study aimed to understand how technical change and efficiency within the French PS fleet fishing in the IO has evolved, given that management mainly targets limited effective fishing effort on the ‘game changer’ use of DFADs since the early 1990s. Neglecting technical change can lead to misleading policy advice for the reduction of fishing capacity and efforts to reduce fishing mortality. For instance, fishing fleets may have modernised their vessels over time via government subsidies or private investment in technology progress. These improvements can result in excess harvesting power within the fleet and thus undermine any fishing capacity or effort reduction programs^41,42^. With the stock biomass levels in the IO for yellowfin and skipjack declining since the 1950s (Fig. 1), it is noticeable that PS effort for the French fleet has increased through the number of FOB sets (Fig. 1). The effect may lead to an excess in fishing capacity, resulting in a race for fish and increasing returns to scale, with investment in larger-sized and power-enhanced vessels to achieve this.

Consequently, the study results show some interesting findings, for instance, regarding the technical change over time via the cumulative chained analysis in Fig. 3a—FOB fishing. Observations from this figure show a dramatic technical change around 2008/2009, when there was a peak in the use of satellite buoys^15^. Since 2010, DFADs were first equipped with echosounder buoys^43^ and were further improved by technology companies throughout the studied time series^23^. The rate of technical change for this fishing strategy was estimated at 8.0% per annum since 2007 and at 3.6% per annum over the entire period. When considering the EC for FOBs in Fig. 3, or how much the fishers can produce given the current technology available, it is apparent that there has been very little change over time, and EC is consistently high and homogenous (Fig. 2b supports this). In other words, productivity growth has been purely driven by advances in technical change.

Considering the rapid advancement of DFAD technology, there are ongoing discussions among the global tuna Regional Fishery Management Organisations (RFMOs) on how to control the use of DFADs and set the quantitative limits each PS vessel can monitor at any given time (IOTC resolution 19/02). Since fleet capacity (number of vessels/or vessel power) is not capped, DFAD management becomes more complex. Therefore, a limit to the capacity/efficiency of the fleet may have to be set. Current measures within IOTC (Res. 19/02) set limits on 300 active instrumented buoys at any one time and no more than 500 acquired annually for each PS vessel. However, with the increased FOB monitoring and technology, less capital stock is needed in production. To assess the factors that influence UCU, it is unsurprisingly evident that the increase in the number of sets on DFADs/FOBs increases output, i.e. for a 1% increase of FOB sets (2013–2019 model), a 0.29% increase in catch is estimated (Table 2). However, a 1% increase in DFAD deployments by each vessel within a year has a small negative effect on UCU. In other words, if FOB deployments were to increase consistently, it could lead to excess fishing capacity and a reduction in UCU. The excess fishing capacity represents underutilised fishing capacity, which can pressure the stocks and economic waste^6^. When examining capacity metrics, here vessel power, the analysis in Table 2 implies that the fishery could become over-capitalised with increased fishing power. These results are as expected; for example, the low disproportionate change in UCU for vessel power (capital input) reflects low intensity in the production process, and a lot of the power is essentially unproductive, in contrast to the number of FOB sets that have a higher impact in the production process. Nevertheless, the average UCU appears to be consistently high ~ 0.75 throughout the period due to the high TE (efficient use of the inputs) (Fig. 2c), which implies these PS are operating fairly efficiently despite the lower capacity utilisation scores.

There could be opportunities via effort (input) controls to restrict capacity to balance capacity with allocated quotas. Management could be achieved via some form of composite measure like, for example, kW-days while also considering the annual technical change and the relationships found here between the total number of sets and catches. Regulating set number limits on FOBs/DFADs, for example, may also go some way to controlling FOB/DFAD fishing as this would be an incentive for fishers to become more selective on the objects they fish on, i.e. catching on larger schools^44^. Regarding the current yellowfin quota measure, the negative relationship with UCU is further evidence of overcapacity in the fishery.

In contrast to the FOB fishing strategy, it is essential to note the changes within the FSC strategy, particularly the concurrent increase of efficiency and technical change over time (Fig. 3b). Figure 3b depicts a lag effect occurring via a series of peaks and troughs concerning an increase in TC followed by an increase in EC. The change in efficiency may represent a “learning by doing” effect, where the implementation of new technologies takes time to perfect for skippers, as stated by authors^45,46^. The incremental dynamics of TE and TC can also be explained by the changing nature of the exogenous factors^41^ (prices^32^, costs^36^, environmental conditions^38,47,48^ and piracy events^37^) far more than the technology itself (see Table 2). However, it is essential to note the fall in fishing effort for the FSC strategy in 2018 (Fig. 1), reflecting changes in the fleet strategy after implementing the yellowfin TAC in 2017 to avoid overshooting the yellowfin quota (Laurent Floc’h *pers comm*).

The models developed here can show the impact of increasing/decreasing set numbers for the two fishing strategies (FOB and FSC) on potential catches and, hence, revenues for the fleets. One of the main findings was the significant increases in efficiency for both strategies throughout time. An assumption in fisheries stock assessment models is that cpue are related to stock abundance. However, due to the aggregating nature of tuna, efficiency and technical changes could increase or maintain cpue, even when the stock declines^30^. This new information on efficiency change and technical change within the French PS fleet could usefully help stock assessment experts to better account for differences in fishing effort dynamics in their models and projections. The models developed in this study were solely based on the French data, representing one-third of the IO PS fleet. Therefore, future assumptions and interpretations of the results should always be made with caution. For instance, what assumptions about the PS fleet need to be made and what data is missing for a more accurate estimation of the fishing mortality affected by technical change and efficiency (e.g., the number of supply boats or investment in new FAD technology).

This study progresses our knowledge of PS productivity and its origin in the IO. It may provide helpful insight for using input/output-based management measures towards the challenging long-term objectives of sustainable tropical tuna fisheries, and further ensure the viability and profitability of both artisanal and industrial fishing fleets that exploit them.

## Methods

### French PS fleet data

Logbook landings data in the IO were obtained from the French Observatory of Exploited Tropical Pelagic Ecosystems (Ob7). The vessel level data for the French PS included catch (e.g. yellowfin and skipjack tuna) and effort data (days fished on FOB and FSC, number of sets by type of fishing strategy—free schools or associated schools) for the years spanning 1992–2019 (see Fig. 1). The vessel characteristic data for the same year period was obtained from a vessel register file also provided by Ob7, which included the number of DFAD deployed with echosounder buoys (2013–2019) for all French PS vessels fishing in the IO. The number of DFADs variable was not used in the more extended time series due to the absence of echosounder buoy data in earlier years because of less advanced technology and fewer data collected and stored. The fleet register contains information on vessel characteristics such as engine power (kW), gross tonnage (GT), and vessel length overall (m). Both data series were combined by year and vessel identification number, and a database was produced (see Table 3).

**Table 3:** Source and description of variables used in the model(s).

Variables	Type	Description	Source
TAC	Factor	1 or 0 (1 = TAC introduced for period 2017–2019)	NA
fadsets	Integer	Number of FOB sets per year	Ob7, Sete, France
fuel	Continuous	Average inflation adjusted to 2015 price of oil per barrel per year in USD	www.eia.gov
yft/skj	Continuous	Catch of yellowfin/skipjack in tonnes	Ob7, Sete, France
events	Integer	Number of piracy events as a measure of intensity	https://msi.nga.mil/Piracy
yftpr/skjpr	Continuous	Average inflation adjusted to 2015 price of yellowfin/skipjack per tonne per year in USD	https://www.customs.go.th/index.php?lang=en&
kW	Continuous	Vessel specific power in kilowatts	Ob7, Sete, France
dmi	Continuous	Indian Ocean Dipole index	https://sealevel.jpl.nasa.gov/data/vital-signs/indian-ocean-dipole/
fscsets	Integer	Number of FSC sets per year	Ob7, Sete, France
buoys	Integer	Maximum number of buoys deployed by vessel by year	Ob7, Sete, France
length	Continuous	Vessel overall length in metres (m)	Ob7, Sete, France
days fishing	Integer	Total number of days fishing by FOB/FSC	Ob7, Sete, France
skj_biomass	Continuous	Total spawning stock biomass of skipjack tuna in tonnes	https://iotc.org/
yft_biomass	Continuous	Total spawning stock biomass of yellowfin tuna in tonnes	https://iotc.org/

### Data envelopment analysis (DEA)

Data Envelopment Analysis (DEA) is a non-parametric method (see ^49^ and ^50^) that can be used to assess the potential output (what is harvested relative to what theoretically could be harvested given the available means) of a Decision Making Unit (DMU) (a fishing vessel in the present study). The method assumes the production function (how outputs change with inputs) is unknown and compares each DMU against all other DMUs^51^. The approach identifies the “frontier” (or envelope), which represents the most efficient combination of various input and output variables for the DMU in question^52^. All else being equal, any DMU of similar characteristics should be able to achieve the same level of output. A DMU lying on the frontier is given a value of one because it is considered efficient. In contrast, a DMU with the same characteristics but a lower output is considered inefficient and has less than one score, revealing inefficiency. The process is deterministic and produces an efficiency score for that DMU.

The inputs for the DEA are selected across fixed and variable effort features. Fixed inputs are vessel engine power/gross tonnage/length overall, representing vessel capital stock (only one is chosen as they are all positively correlated). Variable inputs may include the number of days fished on FOB/FSC and the number of sets on FOBs or free schools within a year. The two outputs were exploitation rates, calculated as annual catches of yellowfin and skipjack tuna by vessel measured in tonnes divided by their respective spawning stock biomass. DEA efficiency scores are calculated annually for each vessel relative to the other vessels in the French PS fleet at that time. We incorporated spawning stock biomass for each species as an exploitation rate over time as an output (e.g. catch/spawning stock biomass). Average scores for the whole fleet are then calculated for each fishing strategy (FSC and FOB) by year. Although different fixed and variable input combinations could be used, we settled for a simple design to evaluate the number of positive sets and buoy deployments and the effect of vessel engine power on capacity utilisation in the second stage of DEA (see Section “Second Stage—Factors That Affect Unbiased Capacity Utilisation (UCU)”).

#### Technical efficiency (TE)

We considered the technical efficiency score scalar, $${\theta }_{1},$$ which determines how much catch (production) of each vessel ($$j)$$ can increase for a given quantity of inputs ($${x}_{j,n })$$, for both fixed input (vessel overall length in meters) and variable inputs ($$n$$), (number of days fished on FOB or FSC), to denote the outputs of each species ($$m$$), $${y}_{j,m}$$, (yellowfin catch/yellowfin spawning stock biomass and skipjack catch/skipjack spawning stock biomass for each vessel $$j$$, i.e. the decision making unit—DMU) in an efficient combination (maximum output—catch). *J* is the total number of vessels. Each fishing activity, FSC and FOB are estimated as separate fishing activities and therefore estimated separately using the appropriate inputs of FOB or FSC sets throughout the study. The relative efficiency is calculated using the output-oriented distance function^33,53^:

1 $$\begin{aligned} & Max\theta_{1} \\ & {\text{Subject to}}, \\ & \theta_{1} y_{j,m} \le \mathop \sum \limits_{j = 1}^{J} z_{j} y_{j,m} \quad \forall m \\ & \mathop \sum \limits_{j = 1}^{J} z_{j} x_{j,n } \le x_{j,n} \quad \forall n \\ & \mathop \sum \limits_{j = 1}^{J} z_{j} = 1 \\ & z_{j} \ge 0\quad \forall j \\ \end{aligned}$$

where ${z}_{j}$ corresponds to weighting factor *z,* for vessel *j* (${z}_{j}$ a weighted sum of all vessel outputs within the year, including the vessel itself) measuring the optimal linear combination of frontier observations that give the optimal performance of the DMU in question (or more specifically the distance from the frontier). Each vessel is measured separately for the value of ${\theta }_{1}$ where ${\theta }_{1}{y}_{j,m}$ represent the outputs of each vessel that can increase using ${x}_{j,n}$ inputs (variable and fixed) in a technically efficient combination. Technically efficient output is equal to the production (observed catch of each tuna species) multiplied by ${\theta }_{1}$, where ${\theta }_{1}\ge 1$ is a scalar representing how much each of the DMU's output can be increased relative to the efficient frontier of a group of DMUs within a year by fishing activity, FOB or FSC.

When calculating technical efficiency within the DEA, assumptions about the ‘returns to scale’ (constant -CRS- or variable -VRS-) must be made as this affects the efficiency score. CRS can be explained as an increase in input that causes a proportional increase in output. The VRS assumes that vessels compete in a context of variable returns and should apply when all DMUs do not operate at their optimal size. We assume variable returns to scale (VRS) ${\sum }_{j=1}^{J}{z}_{j}$ $=1$, the change in output can be greater, equal to, or less than the change in input, which is the general approach adopted in fisheries economics, i.e. non-constant returns to scale (see ^51^).

Technical efficiency (Eq. 1) of each PS vessel operating within a year is calculated as follows:

2 $${\text{TE}} = { }\frac{1}{{\theta_{1} }}{ }$$

Vessels, which are the most technically efficient, operate along the frontier boundary ($\mathrm{TE} = 1$). Those that are less efficient operate within it and have a score value of TE < 1.

#### Estimating capacity utilisation (CU) and unbiased capacity utilisation (UCU)

When estimating TE in (Eq. 2), the assumption is that the variable inputs (days fished on FOB/FSC) remain at their observed level. For estimating CU (Eq. 3), the assumption is that a vessel can adjust its variable inputs (e.g. depending on the vessel's activity under analysis, i.e. days fishing on FOB/FSC) to increase its output. This step enables variable inputs to be fully utilised while outputs are constrained by the fixed inputs ($n\in \alpha$) (see Eq. 3), and remain constant, i.e. the vessel length. We can then calculate the CU by fitting a similar model as in Eq. 1, but by relaxing the bounds of the sub-vector of the variable inputs $n\in \widehat{\alpha }$ (these inputs are left unconstrained—(Eq. 3), where ${\lambda }_{j,n}$ is the input utilisation rate by vessel *j* of fixed input *n*. The assumption is that the capacity output (catch composition) ${\theta }_{2}{y}_{j,m}$ level remains constant. However, the capacity level can increase through different uses of the variable inputs^33^ (see Eq. 3):

3 $$\begin{aligned} & Max\,\theta_{2} \\ & {\text{Subject to}}, \\ & \theta_{2} y_{j,m} \le \mathop \sum \limits_{j = 1}^{J} z_{j} y_{j,m} \quad \forall m \\ & \mathop \sum \limits_{j = 1}^{J} z_{j} x_{j,n } \le x_{j,n} \quad n \in \alpha \\ & \mathop \sum \limits_{j = 1}^{J} z_{j} x_{j,n } \le \lambda_{j,n} x_{j,n} \quad n \in \hat{\alpha } \\ & \mathop \sum \limits_{j = 1}^{J} z_{j} = 1 \\ & z_{j} \ge 0\,\lambda_{j,n} \ge 0\quad n \in \hat{\alpha } \\ \end{aligned}$$

where ${\theta }_{2}\ge 1$ is, a scalar representing how much each DMU's output can be increased relative to the efficient frontier of a group of DMUs within a year. Capacity Utilisation (CU) (Eq. 4) of each PS vessel operating within a year is calculated as:

4 $${\text{CU}} = { }\frac{1}{{\theta_{2} }}{ }$$

Like TE, CU also has an estimated value between 0 and 1. The Capacity Utilisation (CU) measure may be negatively biased because the observed output may not necessarily be produced in a technically efficient manner (see TE in Eq. 1). TE and fishing capacity may deviate from the frontier due to inefficiency or underutilisation. Therefore, separating the effects and estimating unbiased capacity utilisation (UCU) is necessary. The bias can be corrected by combining results from the technical efficiency models (Eqs. 1 and 2) and the capacity utilisation models (Eqs. 3 and 4). The levels of output that can technically be achieved are calculated as unbiased (UCU) by using:

5 $${\text{UCU}} = {\text{CU}}/{\text{TE}}$$

The DEA linear programming model developed in R software benchmarking^54^ was used to implement the above analysis. All individual vessel efficiency scores were averaged by year and fishing strategy (FSC and FOB) and plotted as a time series.

#### Malmquist indices

From the DEA scores (Eq. 1), the Shephard’s output orientated distance index ${\mathrm{d}}_{0}^{\mathrm{t}}($*x*^t^, *y*^t^) is an alternative name for the previous models shown above in Eq. 1^55^. The DMU (or vessel) referred to here as ${d}_{0}^{\mathrm{t}}$ uses inputs *x*^t^ and produces outputs *y*^t^ within time t*,* and $\theta$ is the technical efficiency score scalar (Eq. 1). The DMU’s linear program can be written more generally as follows ${d}_{0}^{t}($*x*^t^, *y*^t^) $\equiv \mathrm{inf}\{\theta >0|\left( {x}^{\mathrm{t}},{\theta }^{-1}{\gamma }^{\mathrm{t}}\right)\in \mathrm{P}\}$, where P is the production set or technically efficient output for a given input set. The productivity change (PC) between two adjacent periods can be calculated as the change between a particular DMU ‘d_0_’ in the two adjacent periods ${\mathrm{d}}_{0}^{\mathrm{t}}($*x*^t^, *y*^t^) and ${\mathrm{d}}_{0}^{\mathrm{t}+1}($*x*^t+1^, *y*^t+1^). It is important to note that the dataset must have a balanced design; therefore, vessels (DMU) must always be present in successive years. This was performed piecemeal-wise as not all vessels were present in the same periods due to vessels entering and exiting the fishery at different periods. The index can be further decomposed into efficiency change (EC) and technical change (TC) following^53^.

6 $$\begin{gathered} {\text{EC}}\quad {\text{TC}} \hfill \\ {\text{PC}}\left( {x^{{{\text{t}} + 1}} ,y^{{{\text{t}} + 1}} ,x^{{\text{t}}} ,y^{{\text{t}}} } \right) = \frac{{{\text{d}}_{0}^{{{\text{t}} + 1}} \left( {x^{{{\text{t}} + 1}} ,y^{{{\text{t}} + 1}} } \right)}}{{{\text{d}}_{0}^{{\text{t}}} \left( {x^{{\text{t}}} ,y^{{\text{t}}} } \right)}}\sqrt {\frac{{d_{0}^{t} \left( {x^{{\text{t}}} ,y^{{\text{t}}} } \right)}}{{{\text{d}}_{0}^{{{\text{t}} + 1}} \left( {x^{{\text{t}}} ,y^{{\text{t}}} } \right)}}.\frac{{{\text{ d}}_{0}^{{\text{t}}} \left( {x^{{{\text{t}} + 1}} ,y^{{{\text{t}} + 1}} } \right)}}{{{\text{ d}}_{0}^{{{\text{t}} + 1}} \left( {x^{{{\text{t}} + 1}} ,y^{{{\text{t}} + 1}} } \right)}}} \hfill \\ \end{gathered}$$

If PC values are > 1, then there have been productivity improvements; on the other hand, if PC < 1, a decline in productivity is observed. TC is the frontier shift between the two periods, e.g.$\mathrm{t}$ and $\mathrm{t}+1,$ which measures the technical improvements due to innovations, and EC is a ratio of the d_0_ of the observation between $\mathrm{t}$ and $\mathrm{t}+1$, coming from a better use of available inputs.

### Second stage—factors that affect unbiased capacity utilisation (UCU)

To explain differences in the DEA analysis's output efficiency scores and to ascertain what factors may affect efficiency, an Ordinary Least Square (OLS) linear regression model was chosen to explore potential factors, which was concluded as an appropriate method to use, as suggested by Hoff^56^. Understanding the factors that influence efficiency and capacity utilisation is essential in management, as changes in these factors may negatively affect any control implemented. Within the regression, the efficiency scores of DMUs were the dependent variables and a variety of variables taken from the literature (ones that weren't used as inputs in the DEA approach) that could explain the deviations between vessels were examined as predictors^57,58^. The information in a given year included real fuel costs and real prices for both yellowfin and skipjack, large PS vessel-specific attributes such as vessel age or kW power, the maximum number of buoy deployments (2013–2019), and the total number of sets (FSC/FOB 1992–2019 and DFAD sets 2013–2019). Other exogenous events affecting the IO tuna fishery were also considered, such as climatic events (e.g. Indian Ocean Dipole data -DMI- averaged by year) and the number of piracy events on all vessels (obtained via https://msi.nga.mil/Piracy accessed 30 January 2023), which were aggregated by year and combined with vessel specific data before the linear models were fit.

For the sake of model selection by fishing strategy, all model predictors were entered into a comprehensive model, and all possible combinations of available predictor variables were systematically fitted, leading to a selection of five candidate models using the R package glmulti^59^. These models were ranked by their Akaike Information Criterion (AIC), and the model with the lowest AIC score was selected. The best-selected model was then bootstrapped 1000 times to overcome the residuals' heteroscedastic nature to fit with small samples. The resulting parameter estimates were bias-corrected, and the elasticities estimated considering the average change in $x$(e.g.for the reference period under analysis 1992–2019) over the average change in *y* (e.g. for the reference period under analysis 1992–2019) multiplied by the parameter estimate of the regressor in the model (*b*). (Eq. 7) gives the elasticity *E* (e.g. a 1% change in *x* provides a b/*E* % change in *y*):

7 $$E = b\left( {\frac{\Delta x}{{\Delta y}}} \right)$$

## Data Availability

The data supporting this study's findings are available from the corresponding authors upon reasonable request and with the permission of IRD.

## References

[1] FAO. The State of the World Fisheries and Aquaculture. Rome: Food and Agriculture Organization of the United Nations. ISBN: 9789251363645 (2022). 10.4060/cc0461en

[2] GLITNIR. Tuna Seafood Industry Report. Glitnir (2007).

[3] McKinney, R., Gibbon, J., Wozniak, E. & Galland, G. Netting billions 2020: A global tuna valuation. The Pew Charitable Trusts, 36 (2020). www.pewtrusts.org/-/media/assets/2020/10/nettingbillions2020.pdf

[4] FAO. The State of World Fisheries and Aquaculture. Sustainability in action. Rome: Food and Agriculture Organization of the United Nations ISBN: 9789251326923 (2020). 10.4060/ca9229en

[5] Costello C (2020). The future of food from the sea. Nature.

[6] Tidd AN, Rousseau Y, Ojea E, Watson R, Blanchard JL (2022). Food security challenged by declining efficiencies of artisanal fishing fleets: a global country-level analysis. Glob. Food Secur..

[7] Garcia SM, Rosenberg AA (2010). Food security and marine capture fisheries: Characteristics, trends, drivers and future perspectives. Philos. Trans. R. Soc. Lond. B: Biol. Sci..

[8] Hilborn R, Hilborn U (2012). Overfishing: What Everybody Needs to Know.

[9] Blasiak R (2017). Climate change and marine fisheries: Least developed countries top global index of vulnerability. PLoS ONE.

[10] Srinivasan UT, Cheung WWL, Watson R, Sumaila UR (2010). Food security implications of global marine catch losses due to overfishing. J. Bioecon..

[11] World Bank. *The Sunken Billions Revisited: Progress and Challenges in Global Marine Fisheries* 100 (The World Bank, 2017). elibrary.worldbank.org

[12] Gillett, R. Tuna for tomorrow: Information on an important Indian Ocean fishery resource. Smartfish working papers. EU. 55pp (2013).

[13] Torres-Irineo E, Gaertner D, Chassot E, Dreyfus-Leon M (2014). Changes in fishing power and fishing strategies driven by new technologies: the case of tropical tuna purse seiners in the eastern Atlantic Ocean. Fish. Res..

[14] Maufroy A (2017). Massive increase in the use of drifting Fish Aggregating Devices (dFADs) by tropical tuna purse seine fisheries in the Atlantic and Indian oceans. ICES J. Mar. Sci..

[15] Gaertner D (2018). Results achieved within the framework of the EU research project: Catch, effort, and eCOsystem impacts of FAD-fishing (CECOFAD). Collect. Vol. Sci. Pap. ICCAT.

[16] Murua H (2023). Lessons learnt from the first large-scale biodegradable FAD research experiment to mitigate drifting FADs impacts on the ecosystem. Mar. Policy.

[17] Dupaix A (2021). Surface habitat modification through industrial tuna fishery practices. ICES J. Mar. Sci..

[18] Capello M, Rault J, Deneubourg JL, Dagorn L (2022). Schooling in habitats with aggregative sites: The case of tropical tuna and floating objects. J. Theor. Biol..

[19] Moreno G (2016). Fish aggregating devices (FADs) as scientific platforms. Fish. Res..

[20] Baidai Y, Dagorn L, Amande MJ, Gaertner D, Capello M (2020). Machine learning for characterising tropical tuna aggregations under Drifting Fish Aggregating Devices (DFADs) from commercial echosounder buoys data. Fish. Res..

[21] Orue B (2020). Comparing the distribution of tropical tuna associated with drifting fish aggregating devices (DFADs) resulting from catch dependent and independent data. Deep Sea Res. Part II: Top. Stud. Oceanogr..

[22] Delgado de Molina, A., Ariz, J. & Areso, J. Statistics of the purse seine Spanish fleet in the Indian Ocean (1990–2011). IOTC-2012-WPTT-14-19 (2012).

[23] Wain G, Guéry L, Kaplan DM, Gaertner D (2021). Quantifying the increase in fishing efficiency due to the use of drifting FADs equipped with echosounders in tropical tuna purse seine fisheries. ICES J. Mar. Sci..

[24] Griffiths SP, Allain V, Hoyle SD, Lawson TA, Nicol SJ (2019). Just a FAD? Ecosystem impacts of tuna purse-seine fishing associated with fish aggregating devices in the western Pacific Warm Pool Province. Fish Oceanogr..

[25] Fonteneau A, Chassot E, Bodin N (2013). Global spatio-temporal patterns in tropical tuna purse seine fisheries on drifting fish aggregating devices (DFADs): Taking a historical perspective to inform current challenges. Aquat. Living Resour..

[26] Tolotti M, Guillotreau P, Forget F, Capello M, Dagorn L (2022). Unintended effects of single-species fisheries management. Environ. Dev. Sustain..

[27] Dagorn L (2012). Targeting bigger schools can reduce ecosystem impacts of fisheries. Can. J. Fish. Aquat. Sci..

[28] Imzilen T (2022). Recovery at sea of abandoned, lost or discarded drifting fish aggregating devices. Nat. Sustain..

[29] Filmalter JD, Capello M, Deneubourg JL, Cowley PD, Dagorn L (2013). Looking behind the curtain: Quantifying massive shark mortality in fish aggregating devices. Front. Ecol. Environ..

[30] Tidd AN, Reid C, Pilling GM, Harley SJ (2016). Estimating productivity, technical and efficiency changes in the Western Pacific purse-seine fleets. ICES J. Mar. Sci.: J. du Conseil.

[31] Escalle, L., Brouwer, S., Pilling, G. & PNA Office. Estimates of the number of FADs active and FAD deployments per vessel in the WCPO. In *A paper submitted to the 14th Regular Session of the WCPFC Scientific Committee, Busan, Korea* (2018).

[32] Lecomte, M., Rochette, J., Laurans, Y. & Lapeyre, R. Indian Ocean tuna fisheries: Between development opportunities and sustainability issues (2017)*.* https://www.iddri.org/en/publications-and-events/report/indian-ocean-tuna-fisheries-between-development-opportunities-and

[33] Tingley D, Pascoe S (2005). Factors affecting capacity utilisation in English Channel fisheries. J. Agric. Econ..

[34] Tidd AN, Caballero V, Ojea E, Watson RA, García Molinos J (2023). Estimating global artisanal fishing fleet responses in an era of rapid climate and economic change. Front. Mar. Sci..

[35] Felthoven RG, Morrison Paul CJ (2004). Multi-output, non-frontier primal measures of capacity and capacity utilisation. Am. J. Agric. Econ..

[36] Chassot E, Antoine S, Guillotreau P, Lucas J, Assan C, Marguerite M, Bodin N (2021). Fuel consumption and air emissions in one of the world’s largest commercial fisheries. Environ. Pollut..

[37] Chassot, E., Guillotreau, P., Kaplan, D. & Vallée, T. Piracy and tuna fisheries. In C. Norchi, G. Proutière-Maulion et C. Leboeuf (Eds), *Piracy in Comparative Perspective: Problems, Strategies, Laws* Ch. 6 (Pedone et Hart, 2012).

[38] Lan KW, Evans K, Lee MA (2013). Effects of climate variability on the distribution and fishing conditions of yellowfin tuna (*Thunnus Albacares*) in the Western Indian Ocean. Clim. Change.

[39] Naylor R (2021). Blue food demand across geographic and temporal scales. Nat. Commun..

[40] Hilborn R (2020). Effective fisheries management instrumental in improving fish stock status. Proc. Natl. Acad. Sci..

[41] Kirkley J, Paul CJM, Cunningham S, Catanzano J (2004). Embodied and disembodied technical change in fisheries: an analysis of the Sète trawl fishery 1985–1999. Environ. Resour. Econ..

[42] Squires D, Vestergaard N (2013). Technical change and the commons. Rev. Econ. Stat..

[43] EC - European Commission, Executive Agency for Small and Medium-sized Enterprises, Gaertner, D., Grande, M., Pascual, P. *et al.* Catch, effort, and ecosystem impacts of tropical tuna fisheries (CECOFAD II): final report, Publications Office, 2020. 10.2826/621446

[44] Davies TK, Mees CC, Milner-Gulland EJ (2014). The past, present and future use of drifting fish aggregating devices (FADs) in the Indian Ocean. Mar. Policy.

[45] Squires D, Kirkley JE (1999). Skipper skill and panel data in fishing industries. Can. J. Fish. Aquat. Sci..

[46] Squires, D. & Reid, C. Using Malmquist indices to measure changes in total factor productivity of purse-seine vessels while accounting for changes in capacity utilisation, the resource stock and the environment. SCTB17 Working Paper. FTWG-5 (2004).

[47] Marsac, F. Outlook of ocean climate variability in the west tropical Indian Ocean, 1997–2008. Paper IOTC-2008-WPTT-27 prepared for the Indian Ocean Tuna Commission Working Party on Tropical Tunas (2008).

[48] Marsac, F., Le Blanc, J.L. Dynamics of ENSO events in the Indian Ocean: to what extent would recruitment and catchability of tropical tunas be affected? In *Proceedings of the Expert Consultation on Indian Ocean Tunas, 7th session, IOTC, Victoria, Seychelles* 9–14/11/98. IOTC Proceedings 1: 89–101 (1998).

[49] Farrell MJ (1957). The measurement of productive efficiency. J. R. Stat. Soc..

[50] Charnes A, Cooper W, Rhodes E (1978). Measuring the efficiency of decision-making units. Eur. J. Oper. Res..

[51] Cooper WW, Seiford LM, Tone K (2000). Data Envelopment Analysis: A Comprehensive Text with Models, Applications, References, and DEA-Solver Software.

[52] Greene, W. H. Frontier production functions. EC-93-20. Stern School of Business, New York University (1993).

[53] Färe R, Grosskopf S, Lovell CAK (1993). Production Frontiers.

[54] Bogetoft P (2012). Performance Benchmarking: Measuring and Managing Performance.

[55] Shephard RW (1970). Theory of Cost and Production Functions.

[56] Hoff A (2007). Second stage DEA: Comparison of approaches for modelling the DEA score. Eur. J. Oper. Res..

[57] Pascoe S, Coglan L, Mardle S (2001). Physical versus harvest-based measures of capacity: the case of the United Kingdom vessel capacity unit system. ICES J. Mar. Sci..

[58] Tingley D, Pascoe S, Coglan L (2005). Factors affecting technical efficiency in fisheries: stochastic production frontier versus data envelopment analysis approaches. Fish. Res..

[59] Calcagno V, de Mazancourt C (2010). glmulti: An R package for easy automated model selection with (Generalized) linear models. J. Stat. Softw..

